# Sepsis-associated acute kidney injury: recent advances in enrichment strategies, sub-phenotyping and clinical trials

**DOI:** 10.1186/s13054-024-04877-4

**Published:** 2024-03-21

**Authors:** Matthieu Legrand, Sean M. Bagshaw, Pavan K. Bhatraju, Azra Bihorac, Ellen Caniglia, Ashish K. Khanna, John A. Kellum, Jay Koyner, Michael O. Harhay, Fernando G. Zampieri, Alexander Zarbock, Kevin Chung, Kathleen Liu, Ravindra Mehta, Peter Pickkers, Abigail Ryan, Juliane Bernholz, Laura Dember, Martin Gallagher, Patrick Rossignol, Marlies Ostermann

**Affiliations:** 1 Division of Critical Care Medicine, Department of Anesthesia and Perioperative Care, UCSF, 521 Parnassus Avenue, San Francisco, CA 94143 USA; 2 Department of Critical Care Medicine, Faculty of Medicine and Dentistry, University of Alberta and Alberta Health Services, Edmonton, Canada; 3 Division of Pulmonary, Critical Care and Sleep Medicine, University of Washington, Seattle, USA; 4 Kidney Research Institute, University of Washington, Seattle, USA; 5 Department of Medicine, University of Florida, Gainesville, FL USA; 6 Intelligent Critical Care Center (IC3), University of Florida, Gainesville, FL USA; 7 Department of Biostatistics, Epidemiology, and Informatics, University of Pennsylvania Perelman School of Medicine, Philadelphia, USA; 8 Department of Anesthesiology, Section on Critical Care Medicine, Wake Forest University School of Medicine, Winston-Salem, NC USA; 9 Outcomes Research Consortium, Cleveland, OH USA; 10 Perioperative Outcomes and Informatics Collaborative, Winston-Salem, NC USA; 11 Department of Critical Care Medicine, University of Pittsburgh, Pittsburgh, PA USA; 12 University Section of Nephrology, Department of Anesthesiology, Intensive Care Medicine and Pain Medicine, Department of Medicine, University of Chicago, Chicago, IL USA; 13 Clinical Trials Methods and Outcomes Lab, Department of Biostatistics, Epidemiology, and Informatics, PAIR (Palliative and Advanced Illness Research) Center, Perelman School of Medicine, University of Pennsylvania, Philadelphia, PA USA; 14 Hospital Münster, Münster, Germany; 15 SeaStar Medical, Denver, CO USA; 16 Divisions of Nephrology and Critical Care Medicine, Departments of Medicine and Anesthesia, University of California San Francisco, San Francisco, CA USA; 17 Department of Medicine, University of California, San Diego, USA; 18 Intensive Care Medicine, Radboudumc, Nijmegen, The Netherlands; 19 Chronic Care Policy Group, Division of Chronic Care Management, Center for Medicare and Medicaid Services, Center for Medicare, Baltimore, MD USA; 20 AM-Pharma, Stadsplateau 6, 3521 AZ Utrecht, The Netherlands; 21 Renal-Electrolyte and Hypertension Division, Department of Medicine, Department of Biostatistics, Epidemiology and Informatics, Center for Clinical Epidemiology and Biostatistics, Perelman School of Medicine, University of Pennsylvania, Philadelphia, PA USA; 22 The George Institute for Global Health, University of New South Wales, Sydney, Australia; 23 FCRIN INI-CRCT (Cardiovascular and Renal Clinical Trialists), Nancy, France; 24 INSERM CIC-P 1433, CHRU de Nancy, INSERM U1116, Université de Lorraine, Nancy, France; 25 Medicine and Nephrology-Hemodialysis Departments, Monaco Private Hemodialysis Centre, Princess Grace Hospital, Monaco, Monaco; 26 Department of Critical Care, King’s College London, Guy’s & St Thomas’ Hospital, London, UK

## Abstract

Acute kidney injury (AKI) often complicates sepsis and is associated with high morbidity and mortality. In recent years, several important clinical trials have improved our understanding of sepsis-associated AKI (SA-AKI) and impacted clinical care. Advances in sub-phenotyping of sepsis and AKI and clinical trial design offer unprecedented opportunities to fill gaps in knowledge and generate better evidence for improving the outcome of critically ill patients with SA-AKI. In this manuscript, we review the recent literature of clinical trials in sepsis with focus on studies that explore SA-AKI as a primary or secondary outcome. We discuss lessons learned and potential opportunities to improve the design of clinical trials and generate actionable evidence in future research. We specifically discuss the role of enrichment strategies to target populations that are most likely to derive benefit and the importance of patient-centered clinical trial endpoints and appropriate trial designs with the aim to provide guidance in designing future trials.

## Introduction

Acute kidney injury (AKI) is associated with serious short- and long-term complications [1]. Critically ill patients with severe AKI [defined as Kidney Disease: Improving Global Outcomes (KDIGO) AKI Stage 2 or 3] have an in-hospital mortality greater than 25% which exceeds 50% when renal replacement therapy (RRT) is needed. In the PROCESS study, 60-day hospital mortality was 6.2% for patients without AKI, 16.8% for those with stage 1 and 27.7% for patients with AKI stage 2 or 3 [2, 3]. Patients with less severe AKI, including subclinical AKI [defined as early kidney damage identified by biomarkers without serum creatinine (SCr) rise] are also at risk of both short- and long-term complications, including incident or worsening chronic kidney disease (CKD) and major adverse cardiovascular events (MACE) [4–6].

Sepsis is the most common contributing factor to AKI in acutely and critically ill patients [7]. Our understanding of the pathophysiology of sepsis-associated AKI (SA-AKI) has improved over the last few years and promising therapeutic targets are emerging [8], giving hope to improved clinical outcomes. The 2023 Kidney Disease Clinical Trialists (KDCT) workshop, held in Washington D.C. (USA) in March 2023, provided multiple stakeholders, including clinical researchers, regulatory authorities and commercial partners, with a scientific forum to discuss the current state of SA-AKI clinical research, identify challenges and priorities, and propose strategies for future research toward precision medicine (Table 1) [9]. The scientific program was developed by the KDCT scientific academic committee and focused predominantly on the role of sub-phenotying, enrichment strategies, selection of appropriate endpoints and outcomes, and consideration of alternative trial designs. This narrative review summarizes the presentations, discussions and conclusions of the meeting but does not include a systematic review of the existing literature.

**Table 1:** Description of the terminology used in precision medicine, adapted from Seymour et al. [64] and Stanski et al. [65]

Term	Description
Phenotype	Clinical features or traits that characterize a group of patients within a disease or syndrome, including genetics, environmental factors and other clinically observed characteristics
Endotype	Subset of patients defined by distinct biological mechanism of disease within a phenotype
Sub-phenotype	A subset of clinical features in patients with a shared phenotype that distinguishes the group from other groups within that phenotype
Prognostic	Indicators used to inform about risks of various outcomes
Predictive	Indicators providing information about the likelihood of response to a given treatment
Drug (or intervention) response	Differential responses to drug (or intervention) based on phenotype defined by an indicator
Heterogeneity of treatment effects (HTE)	Differences in treatment responses in a group due to variability in drug response phenotype within that group
Treatable trait	A subgroup characteristic that can be successfully targeted by an intervention
Enrichment	A prospective strategy for addressing HTE by reducing heterogeneity of the sample population or increasing representation of patients with similar risk profiles

## Why should SA-AKI be considered a specific entity?

The reported occurrence rate of SA-AKI varies between 25 and 75% depending on the patient cohort, type and severity of sepsis, and criteria used to define the condition [8]. The prognosis is variable but current data suggests that SA-AKI is associated with a higher risk of mortality and a lower chance of renal recovery than other types of AKI.

In 2022, an international Acute Disease Quality Initiative (ADQI) consensus meeting focused on the definition, epidemiology and management of SA-AKI [8]. The expert panel acknowledged that SA-AKI was a heterogeneous syndrome that occurs as a direct consequence of sepsis (i.e., sepsis-induced AKI) or as a result of indirect mechanisms driven by interventions for sepsis or, in rare cases, because of factors not directly related to sepsis but nevertheless occurring in these patients. In the absence of an accepted definition of SA-AKI, the panel proposed to define SA-AKI by the presence of both, sepsis (as per Sepsis-3 criteria) and AKI (as defined by the KDIGO criteria). Further, sepsis-induced AKI was considered a sub-phenotype of SA-AKI in which sepsis-induced mechanisms directly lead to kidney damage.

**Fig. 1:**
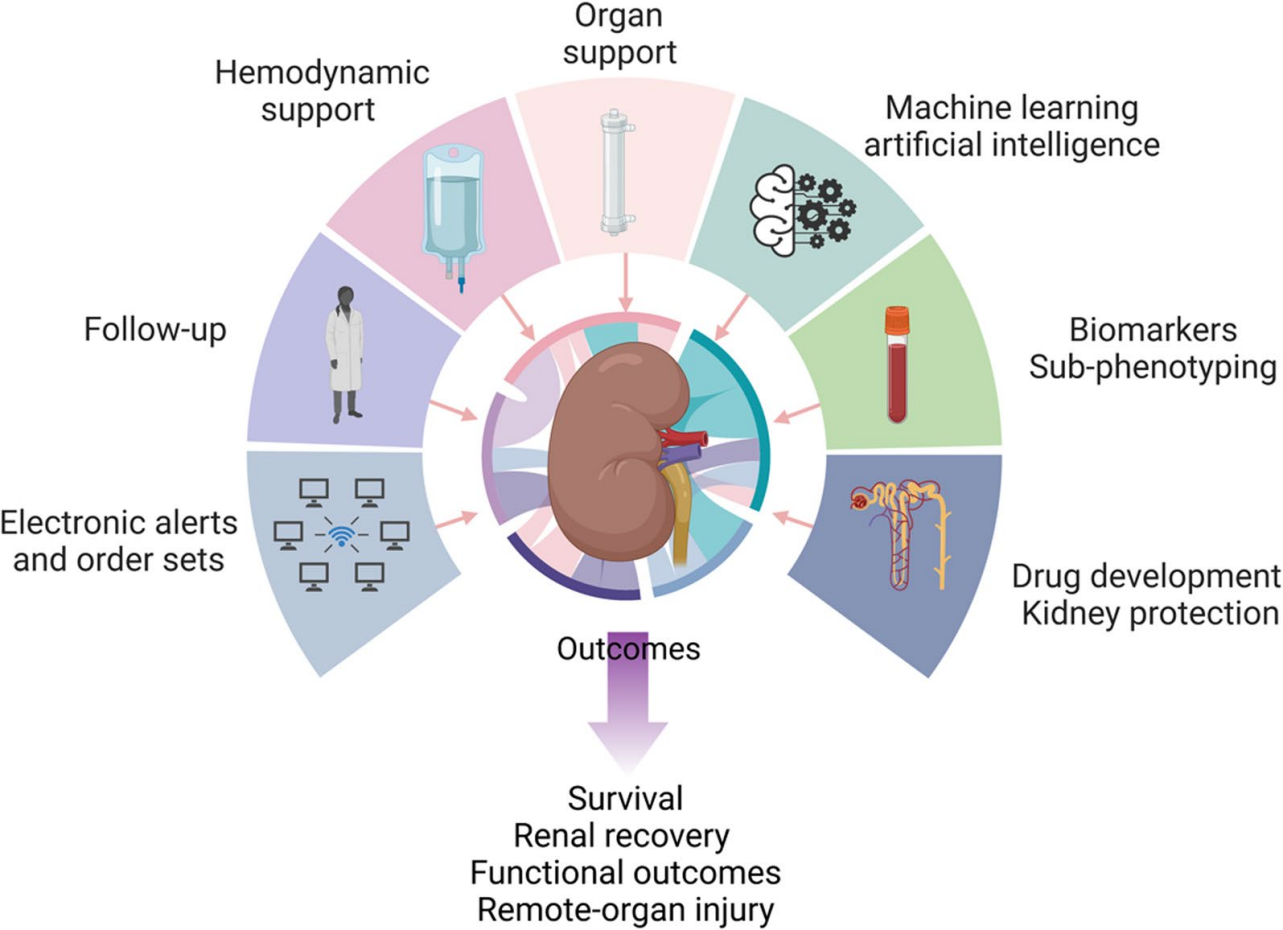
Areas identified as potential candidates to improve sepsis-associated AKI outcomes and to be considered as priorities for testing in clinical trials

The understanding of the pathophysiology of SA-AKI has significantly improved thanks to advances in experimental model design and analytical techniques. Several specific processes and mechanisms have been identified that may contribute to the development of glomerular dysfunction and/or tubular injury in sepsis [7, 10]. These include endothelial dysfunction, inflammation, alteration of the renal microcirculation, activation of the renin–angiotensin–aldosterone system (RAAS), mitochondrial dysfunction, complement activation, direct tubular injury and metabolic reprogramming [11]. While an in-depth review of the pathophysiology of SA-AKI is beyond the scope of this review, a few key processes believed to be important contributors are listed as they may be targets for therapeutic interventions (Fig. 1).

## Role of biomarkers for sub-phenotyping and enrichment

The application of various different biomarkers has provided granularity to the syndrome of AKI and allowed the identification of sub-phenotypes with different etiologies, pathophysiological mechanisms and outcomes. One of the benefits is the opportunity for prognostic and predictive enrichment in clinical trials. Prognostic enrichment describes the application of a biomarker to identify a cohort of patients that is at high risk for a specific outcome (e.g., severe persistent AKI, advanced CKD, mortality), whereas predictive enrichment aims to identify patients who are likely to respond in a similar way to a specific treatment, usually because they share a common underlying pathobiology [12].

### Prognostic enrichment sub-phenotyping

Identifying SA-AKI sub-phenotypes with a higher risk of poor outcome provides opportunities to target interventions toward the higher risk group and to exclude cohorts that may not benefit or potentially come to harm. Two recent studies by Ozrazgat-Baslanti et al. tracked the clinical trajectories of AKI, one for surgical patients and one for all hospitalized patients with AKI [13, 14]. For surgical patients, the ADQI criteria were used to differentiate between ‘No AKI,’ ‘Rapidly Reversed AKI’ ‘Persistent AKI with Renal Recovery,’ and ‘Persistent AKI without Renal Recovery’ [14]. Surgical patients with sepsis who exhibited ‘Persistent AKI without Renal Recovery’ had the highest hospital mortality (45%), RRT use (40%) and decline of glomerular filtration rate (GFR) in the year following surgery. Among hospitalized patients, those with ‘Persistent AKI without Renal Recovery’ also had the highest hospital mortality (28%), need for RRT (13%) and risk of death within one year of discharge (19%). For clinicians and researchers, the challenge is to identify these at-risk patients early and to investigate potential interventions that may modify any of the outcomes.

Among patients included in the FROG-ICU and ADRENOSS study, the authors showed that an elevated proenkephalin level > 80 pmol/L (found in roughly 6% of the cohorts) at the time of admission to the Intensive Care Unit (ICU) without meeting the serum creatinine (SCr) or urine output criteria of AKI, was associated with an increased risk of mortality [15, 16]. In a planned sub-study of the PROCESS trial (a randomized controlled trial exploring the role of early goal directed therapy), the authors measured urinary cell cycle arrest markers tissue inhibitor of metalloproteinases 2 (TIMP-2) and insulin-like growth factor binding protein 7 (IGFBP7) before and after the 6-hour resuscitation period [17, 18]. They demonstrated that patients who still had an elevated biomarker level ([TIMP-2]·[IGFBP7] > 0.3) after receiving fluid resuscitation were at higher risk for a composite endpoint of progression to severe AKI (Stage 2/3), receipt of dialysis or mortality. The incidence of this composite endpoint was similar in patients with elevated biomarkers post-resuscitation regardless of their pre-resuscitation biomarker status and also similar in those with and without AKI at enrollment based on SCr and urine output criteria.

Accurate sub-phenotyping based on clinical features will be essential to making full use of the large-scale electronic health record (EHR) data to understand and manage AKI. Confusion can result from existing efforts to define sepsis subclasses, where researchers used different approaches for classification (empirical, hypothesis-based or agnostic) and interchangeable terms (such as subgroup, sub-phenotype or endotype) that were not reconciled with terminology applied in previously published studies [19]. Recent studies in unsupervised machine learning (ML) have given promise to the prospect of classifying AKI patients into sub-phenotypes. These ML models characterize sub-phenotypes without adhering to any preconceived hypothesis or guidelines. Three published studies separately developed ML models to sub-phenotype adult ICU patients with AKI [20–22], adult ICU patients with SA-AKI within 48 h of admission and hospitalized adults with AKI within 48 h of admission. Two studies identified biomarkers that were significantly different between sub-phenotypes, and all studies were able to differentiate sub-phenotypes related to decreased renal function and higher mortality [20, 21, 23]. Finally, AKI trajectories have been proposed to define different phenotypes. The ADQI group published consensus statements regarding the definitions of ‘SA-AKI’ and its timing (‘early SA-AKI,’ within 48 h of diagnosis of sepsis and ‘late SA-AKI,’ defined as AKI between 48 h to day 7 after sepsis diagnosis) and also adopted the previously proposed timelines for AKI (7 days or less), acute kidney disease (1–90 days) and chronic kidney disease (90 + days) [8].

A major challenge is the fact that current methods for data-driven phenotyping are heterogeneous, there are no reproducible approaches across differing methodologies and datasets to identify sub-phenotypes and endotypes, and resources to aggregate the existing strategies for clinical impact are lacking [24]. These models were developed in specific cohorts and settings and their generalizability remains uncertain across different health systems and critical care units. Confirmatory studies in diverse settings will be needed. Any proposed sub-phenotypes should be assessed by testing for (1) consistency and reproducibility in other datasets, (2) biological plausibility and (3) clinical utility (i.e., ability to identify patients at high risk for a specific outcome or to predict treatment response). Finally, the ideal phenotyping algorithm should impact clinical decision making in real time and provide increased value over current severity scores.

### Predictive enrichment sub-phenotyping

**Fig. 2:**
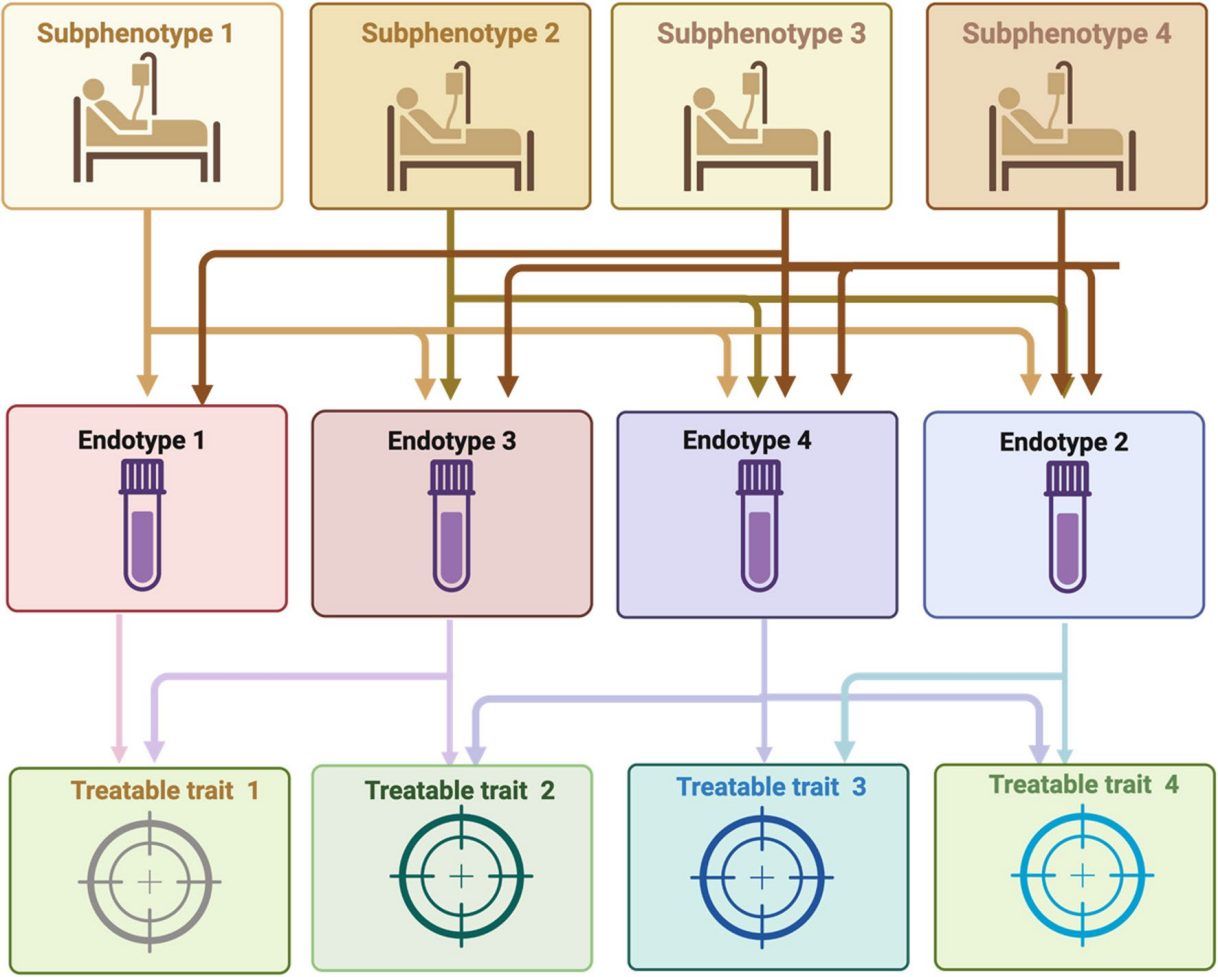
Visual representation of sub-phenotypes (classifying patients based on clinical and physiological characteristics), endotypes (classifying patients based on mechanistic pathways underlying the phenotypes) and treatable traits that would lead to targeted therapies to be tested in randomized trials. Of note, overlap can exist between different phenotypes, endotypes and treatable traits

Because of the heterogeneous nature of critical illness, many clinical studies have not been able to identify treatment benefits. The goal of precision medicine is to match the best available treatment option with the specific patient populations. Developing a classification system for biomarker-driven AKI endotypes will improve the understanding of AKI and enable researchers to engage in more specific therapeutic trials, thus getting closer to the goal of providing precision medicine to patients with AKI [24, 25] (Fig. 2).

In the setting of SA-AKI, an appealing approach is using big data to identify cohorts with shared biology (i.e., comorbidities, laboratory results, clinical variables, biomarkers) [16, 18]. Several groups have used artificial intelligence and advanced ML methods to find a variety of signals in the wealth of available data.

In the FINN-AKI cohort, investigators applied latent class analysis (LCA) and differentiated between two endotypes of AKI in patients with sepsis [21]. Patients with endotype-2, defined by higher plasma levels of inflammatory and endothelial injury markers, had higher 90-day mortality compared to endotype-1 (41% vs 29%) and also a lower probability of short-term renal recovery.

In a retrospective analysis of two ICU cohorts, Bhatraju and colleagues used LCA to characterize two distinct SA-AKI endotypes [26]. All patients had plasma collected within 48 h of ICU admission; 29 different clinical and laboratory values and seven vascular host and inflammatory biomarkers were included in the analysis. Different levels of specific biomarkers (including angiopoietin 1 and 2) and significant differences in the incidence of certain single-nucleotide polymorphism (SNPs) within angiopoietin-2 were identified across the 2 endotypes. A two-group model best separated the data with approximately 60% of patients in endotype-1 and 40% in endotype-2 category. These findings were replicated in a validation cohort where endotype-2 was associated with a 2 or more greater risk of renal non-recovery and 28-day mortality compared to endotype-1, even after adjusting for severity of illness.

The authors then developed a parsimonious prediction model that included the ratio of angiopoietin-2/1 and soluble tumor necrosis factor receptor-1 (sTNFR-1). The model had fairly good c-statistic to predict AKI sub-phenotypes; patients with lower biomarkers of endothelial dysfunction and inflammation were characterized as endotype-1. When applying the model to the VASST database (vasopressin versus norepinephrine in patients with septic shock), the authors observed that these two AKI endotypes identified prior to randomization showed heterogeneity of treatment effect for the early addition of vasopressin to norepinephrine [26]. Specifically, patients classified as endotype-1 had a lower 90-day mortality with the early addition of vasopressin, while mortality was not significantly different in the endotype-2 group if randomized to vasopressin. Further research is necessary to test these endotypes in prospective trials in SA-AKI. Of note, these sub-phenotypes share characteristics with other sub-phenotypes reported in other conditions such as acute respiratory distress syndrome (ARDS) [27]. This observation may not be surprising given that (i) the mechanisms of sepsis-induced organ failure are likely (at least partially) shared between organs, and (ii) similar biomarkers were used to identify sub-phenotypes. In this line, sepsis is the leading cause of ARDS and sub-phenotypes were replicated between sepsis and ARDS [28].

Seymour and colleagues harnessed data from four distinct sepsis cohorts (over 40,000 patients) to develop and validate four distinct phenotypes of sepsis [29]. They demonstrated differences in the incidence of organ injury (AKI, liver failure), as well as differences in biomarkers (inflammatory, coagulopathy, etc.) between sub-phenotypes with cross-variation in sub-phenotypes within trials and the differential treatment effects. These studies suggest that heterogeneity of treatment effect exists across sub-phenotypes and that so-called 'negative' therapies may be reconsidered in enriched SA-AKI populations and among specific sub-phenotypes.

### Predictive enrichment in randomized clinical trials

Most AKI sub-phenotypes were discovered or validated retrospectively using existing databases. In only a few prospective studies and clinical trials, biomarker-based sub-phenotyping was implemented for predictive enrichment. The EUPHRATES trial was an interventional RCT where a biomarker (endotoxin activity) was used to risk stratify patients for enrollment, regardless of AKI status. Patients with an endotoxin activity of 0.6 or higher were randomized to receive two hemoadsorption treatments using polymyxin B and usual care versus sham hemoperfusion and usual care [30]. There was no difference in 28-day mortality between both groups. However, a subgroup analysis identified a ‘sweet spot’ of endotoxin activity (between 0.6 and 0.9) where patients may derive a benefit from hemoadsorption [31]. The trial did not include an assessment of the response to therapy and the protocol had no customization to patients’ response. It could be argued that some patients may have benefitted from greater customization of therapy.

A new trial, Tigris (NCT03901807), is currently underway to test this hypothesis prospectively. Importantly, the statistical analysis plan describes combining Tigris and EUPHRATES data using Bayesian statistics [32]. This approach is similar in concept to adaptive clinical trials which drop certain arms or groups as the trial progresses or even add or eliminate interventions. These adaptations incur less penalty when planned prospectively and may be important tools for future studies in SA-AKI.

In the ATHOS-3 trial [33], patients with vasodilatory shock on high-dose vasopressors were randomized to receive synthetic Angiotensin II or placebo with continuation of other vasopressors. The trial demonstrated that about 70% of patients in the intervention arm met the primary endpoint of a mean arterial pressure (MAP) > 75 mmHg or increase by 10 mmHg or more from baseline within 3 h of drug initiation. While the phase III trial did not show a mortality difference, subsequent work in specific subgroups suggested a survival benefit, including those with septic AKI and patients in whom Angiotensin II was introduced at lower doses of vasopressors [34, 35].

Healthy volunteers have low levels of angiotensin I and angiotensin II and a low angiotensin I/II ratio. In contrast, some patients with vasodilatory shock have very high levels of angiotensin I with a significantly elevated angiotensin I/II ratio, suggesting reduced conversion of angiotensin I to angiotensin II, resulting in angiotensin II deficiency. Patients with a substantially elevated Ang I/Ang II ratio, who were randomized to receiving exogenous Angiotensin II, had a survival benefit compared to the group that received placebo [36]. In a post hoc analysis of the ATHOS-3 trial, the authors measured angiotensin I and renin levels over time (at study initiation and 3 h later) and demonstrated that the angiotensin I and renin concentrations did not decrease in patients who received placebo while the levels fell in those who were randomized to angiotensin II therapy [37]. Based on this finding, the authors hypothesized that the activity of the angiotensin converting enzyme (ACE) was reduced in patients with septic shock and/or endothelial dysfunction [38]. Giving angiotensin II to a subgroup of septic patients with hyperreninemia enrolled in the ATHOS-3 trial was associated with lower mortality. ACE is largely a pulmonary capillary endothelial enzyme, the activity of which decreases with increase in severity of lung injury. It is similarly proposed to decrease in those with other reasons associated with altered pulmonary blood flow, such as cardiac surgical patients or patients on extracorporeal membrane oxygenation (ECMO) [39]. ACE activity is difficult to measure. However, reduced ACE activity usually results in increased renin release and potential diversion of the RAAS pathway through ACE 2, leading to more Ang 1–7 than Ang II [40, 41]. Thus, renin can be considered a surrogate marker of ACE activity. However, a true point of care bedside renin assay to target therapy is lacking and further work to define the role of biomarkers of the RAAS for predictive enrichment to guide Angiotensin II administration is needed. Finally, these post hoc analyses highlight the overlap between sub-phenotypes, have a relative small sample size with a risk of type 1 error and should be considered exploratory (Table 2).

**Table 2:** Examples of different sub-phenotypes derived from post hoc analyses of a randomized controlled trial (Angiotensin II for the Treatment of High-Output Shock-ATHOS-3-trial) [33]

First author (year)	Sub-phenotype 1	Sub-phenotype 2	Main results	Baseline NED
Wieruszewski et al. [34] 3/18/2024 5:35:00 PM	Low NED (≤ 0.25 μg/kg/min) at baseline, *n* = 104	High NED (> 0.25 μg/kg/min), *n* = 217	In patients receiving low NED at randomization, administration of AT II was associated with lower 28-day mortality (HR 0.509; 95% CI 0.274–0.945, *p* = 0.03), while no difference was observed in the high-NED subgroup (HR 0.933; 95% CI 0.644–1.350, *p* = 0.71)	0.21 (0.18–0.23) μg/kg/min in the low-NED group vs 0.47 (0.33–0.68) μg/kg/min in the high-NED group for patients in the AT II group
Bellomo et al. [37]	Low serum renin concentrations at baseline (below median), *n* = 127	High serum renin concentrations at randomization (above median), *n* = 128	In patients with high renin concentrations, treatment with AT II was associated with lower 28-day mortality (50.9%) compared to placebo (69.9%, unstratified hazard ratio, 0.56; 95% confidence interval, 0.35 to 0.88; *p* = 0.012)	0.36 (0.23–0.50) μg/kg/min in the high renin group treated with AT-2 vs 0.40 (0.29–0.69) μg/kg/min in the placebo group
Ham et al. [66]	Patients requiring ≤ 5 ng kg/min angiotensin II at 30 min	Patients receiving > 5 ng/kg/min angiotensin II at 30 min	Day 28 survival was higher in the ≤ 5 ng/kg/min subgroup versus the > 5 ng/kg/min subgroup (59% vs 33%, respectively; hazard ratio, 0.48 [95% CI 0.28–0.72], *p* = 0.0007)	0.52 (0.301) µg/kg/min (SD) in the low dose vs 0.45 (0.377) µg/kg/min (SD) in the high-dose group
Tumlin et al. [35]	Patient receiving RRT at study drug initiation (*n* = 45 AT II, *n* = 60 placebo)	–	In patients receiving RRT at study drug initiation, patients in the AT II group had higher 28-day survival vs placebo (unadjusted HR 0.52; 95% CI 0.30–0.87; *p* = 0.012)	0.46 (0.32–0.78) μg/kg/min in the placebo vs 0.36 (0.23–0.49) μg/kg/min in the AT II group

In the ATHOS-3 trial, 321 patients with vasodilatory shock receiving high doses of vasopressors (> 0.2 µg/kg/min of norepinephrine-equivalent dose) were randomized to receive either angiotensin II or placebo

AT II, angiotensin II; HR, hazard ratio; NED, norepinephrine-equivalent dose; RRT, renal replacement therapy; CI, confidence interval

## Which endpoint for SA-AKI clinical trials?

Clinical trials in critical care, and particularly in critical care nephrology, have traditionally focused on endpoints believed to be of clinical importance such as kidney recovery (i.e., progression to end-stage kidney disease [ESKD] or non-recovery from AKI) [42, 43]. The risk of death is high in critically ill patients, particularly in those with sepsis and multi-organ dysfunction including AKI, approaching 40–50%. For approval of new drugs, regulatory authorities currently favor endpoints such as all-cause mortality or ‘Major adverse kidney event’ (MAKE), a composite of all-cause mortality or receipt of dialysis or significant decline in kidney function [44, 45]. However, death is also an important competing endpoint of renal recovery and may be affected by many other factors than AKI. Even though MAKE considers the competing risk of death with recovery from AKI, the attributable mortality of AKI and non-recovery from AKI is uncertain and a substantial fraction of death may not be related to kidney events. Recovery from AKI is another important endpoint [46]. Non-recovery from AKI is associated with higher mortality and morbidity, including a risk for chronic kidney disease. However, assessing recovery solely using serum creatinine can be misleading in sepsis given the decrease production and increase volume of distribution that may overestimate the renal function. Alternative biomarkers and measuring glomerular filtration rate (i.e., iohexol clearance) may better fit the purpose.

Although not specific to SA-AKI, additional endpoints have been proposed that may also better reflect patient and family perceptions of their experience and outcomes after critical illness [47–49]. These include endpoints representing preservation (or improvement) of their function [e.g., physical function; activities of daily living (ADL), instrumental activities of daily living (IADL)], mental health, cognitive function or health-related quality-of-life (HRQOL)) [50].

**Fig. 3:**
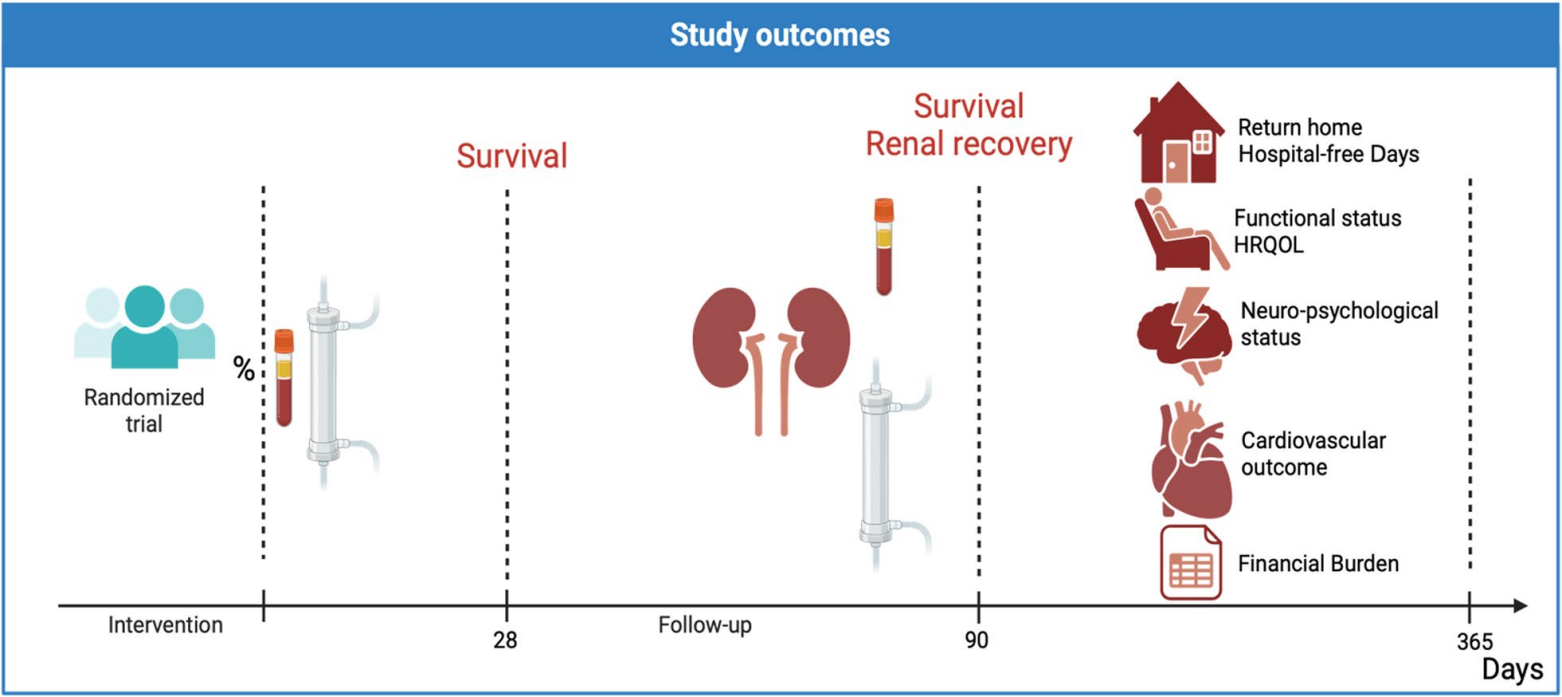
Visual representation of key outcomes to be considered in clinical trials investigating interventions in sepsis-associated AKI

How these endpoints rank in terms of importance to patients and their families is not certain and will need to be explored. Critical illness, major adverse kidney events, chronic kidney failure and longer-term RRT are certainly associated with impaired HRQOL but patients’ preferences differ depending on their personal views, sociocultural impact and circumstances (Fig. 3) [51–53].

Clinical trials have generally not routinely integrated a wider spectrum of survivorship endpoints that may have great (or even greater relative) importance to patients (i.e., disability; return to home, social function; return to work) and their families (e.g., caregiver burden). Ability to return home, number of days at home, freedom from dialysis and hospital-free days were proposed as potential important patient-centered outcomes for patients suffering from critical illness [49, 54].

Few studies have explored measures of the financial burden of critical illness as endpoints, specifically from the patient and family perspective. Financial challenges after critical illness are commonly experienced, particularly in selected health systems and may derive from medical bills, (e.g., cost for dialysis) changes in insurance coverage, and the loss of employment income [7]. Financial concerns are likely very important outcomes and a source of tremendous stress for patients and their families [55]. Moreover, they may be experienced by families long after the death of their family member.

## Innovative clinical trials designs

**Fig. 4:**
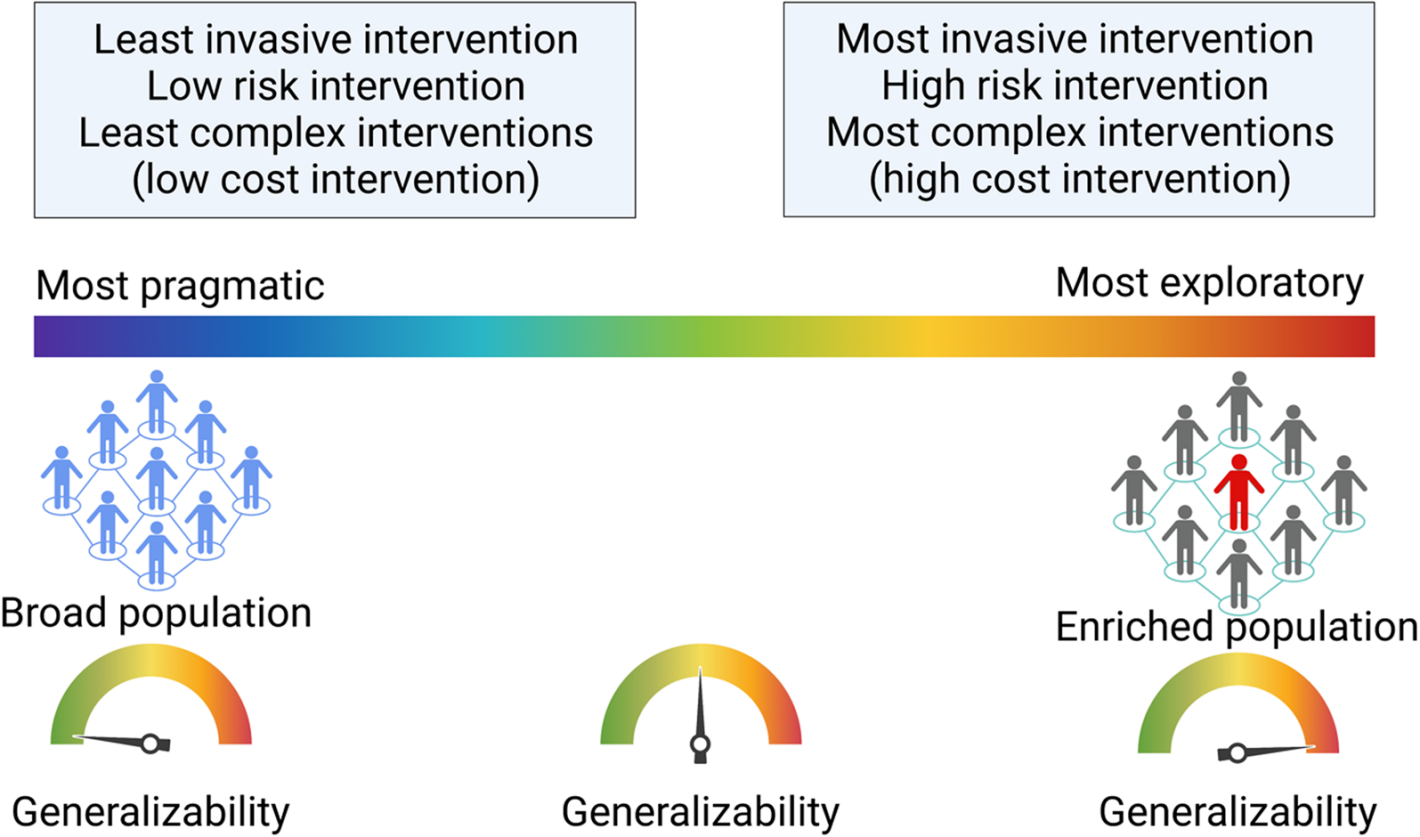
Critical features of an intervention under investigation when designing a more pragmatic or exploratory trial. More pragmatic trials enroll a broader population and tend to increase the generalizability of the trial while more exploratory trials enroll enriched population, at the cost of lower generalizability

Beside classic RCT’s with individual patient randomization, more pragmatic designs have gained popularity over the last years [43, 56] (Fig. 4), including in the field of AKI. Designs such as cluster crossover randomized trials have been applied with success in critically ill patients using renal endpoints (i.e., MAKE30) [57, 58]. The cluster crossover design is an efficient approach particularly for trials in which the intervention can be brief and the endpoints occur within a short period of time. The use of EHR to collect data potentially reduces financial costs and also the potential for errors introduced through data entry by research personnel. Most importantly, cluster crossover trials allow the intervention of interest to be embedded into a clinical care workflow which increases the generalizability of the results, supports sustainability of the intervention after the trial and reduces the cost of implementing the trial.

Platform trials allow the testing of multiple interventions and drugs while using a main master protocol, thus gaining efficiency [59]. A successful example is the SMART trial, in which 15,802 adults were randomized to saline or balanced crystalloids using a cluster crossover design. The incidence of MAKE was 14.3% in the balanced fluid group versus 15.4% in the saline group [odds ratio (OR) 0.90; 95% CI 0.82–0.99] [57]. In a secondary analysis of the subgroup of 1641 septic patients, the balanced crystalloids group had a lower 30-day in-hospital mortality compared to the saline group (26.3% vs 31.2%; adjusted OR, 0.74; 95% CI 0.59–0.93), along with a lower incidence of MAKE and a higher number of vasopressor- and RRT-free days compared to the saline group [57].

Another promising area in clinical trial design involves the a priori assessment of heterogeneity in treatment effects (HTE). Clinical trials estimate the average treatment effect on the included sample. As discussed earlier, it is conceivable that different etiologies and sub-phenotypes of AKI or different population (i.e., women vs men) may respond differently to interventions. The assessment of HTE can be made a priori using cluster/phenotypes or proper methods to estimate individualized treatment effects [60]. Assessments of HTE have been suggested to re-assess several completed trials and may be specified a priori to maximize the validity. In a recent report of the effects of ACE inhibitor and angiotensin receptor blocker (ARB) on COVID-19 patients [61], an individualized treatment effect analysis was designed and reported a priori. Patients were randomized to initiation of an ACE inhibitor (*n* = 257), ARB (*n* = 248), ARB in combination with a chemokine receptor-2 inhibitor (*n* = 10) or no renin-angiotensin-system inhibitor (control; *n* = 264). The investigators estimated the individual-level treatment effect, conditioned on patients’ baseline covariates, using machine learning techniques. Expected absolute risk differences were then calculated for conditional average treatment effects at both the individual and subgroup levels. Although no signal of HTE was observed, other trials have succeeded in finding signals of HTE which could, in theory, be useful for guiding both, clinical practice and determining areas for further studies [62, 63]. Explorations of HTE in AKI trials are needed.

## Conclusions

SA-AKI is associated with very high mortality and morbidity. Over the last several years, various important clinical trials have improved our understanding of SA-AKI and impacted clinical care. Recent advances in sub-phenotyping and clinical trial design offer unprecedented opportunities to generate better evidence in these high-risk patients to improve outcomes.

## Data Availability

Not applicable.
